# Supplementary figures and images for: Enhanced Expression of Glycolytic Enzymes and Succinate Dehydrogenase Complex Flavoprotein Subunit A by Mesothelin Promotes Glycolysis and Mitochondrial Respiration in Myeloblasts of Acute Myeloid Leukemia
Source: Int J Mol Sci. 2024 Feb 10;25(4):2140. doi: 10.3390/ijms25042140 (PMC10888725; doi:10.3390/ijms25042140)

Supplementary Figure S1

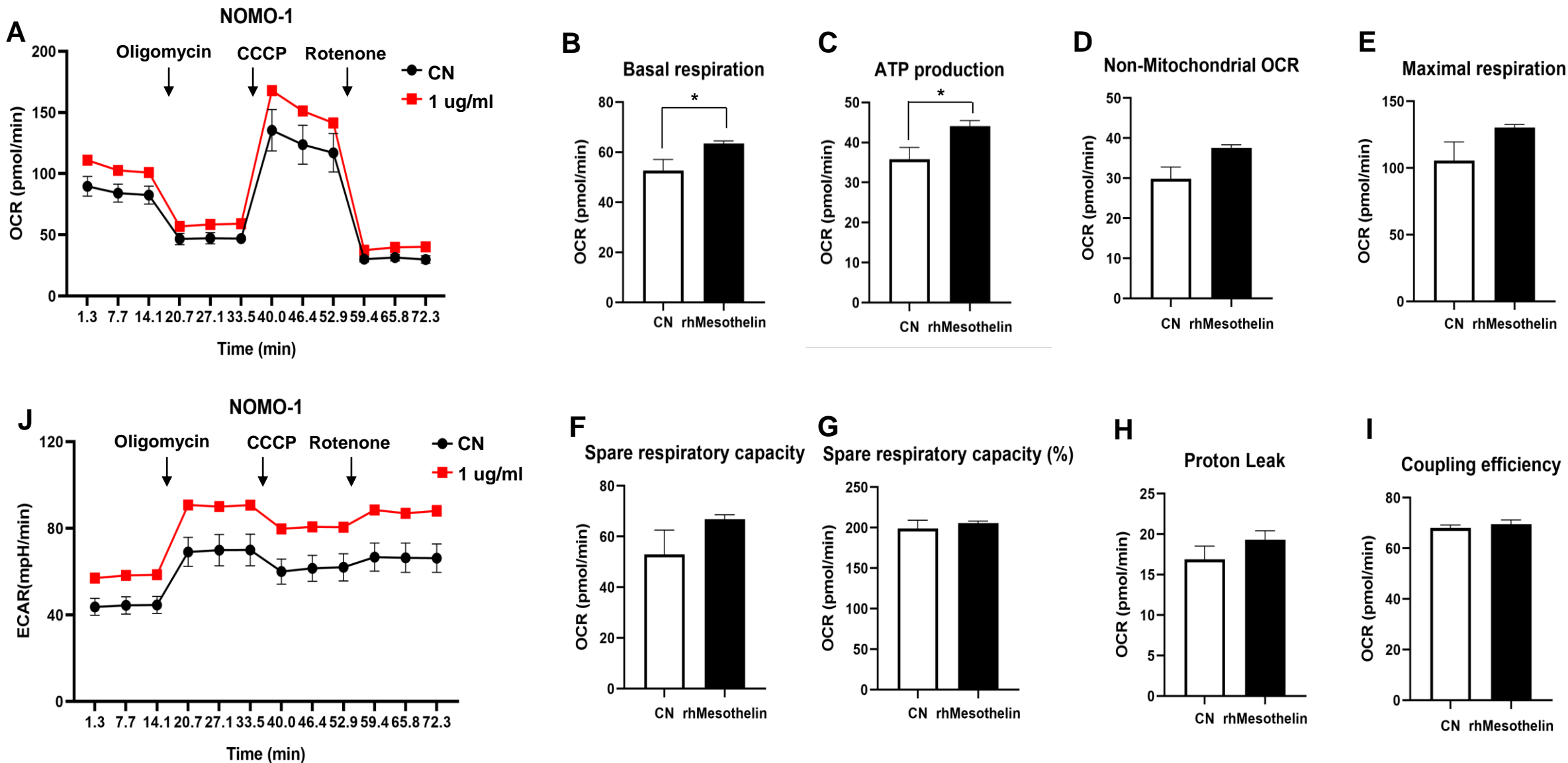

Supplementary Figure S2

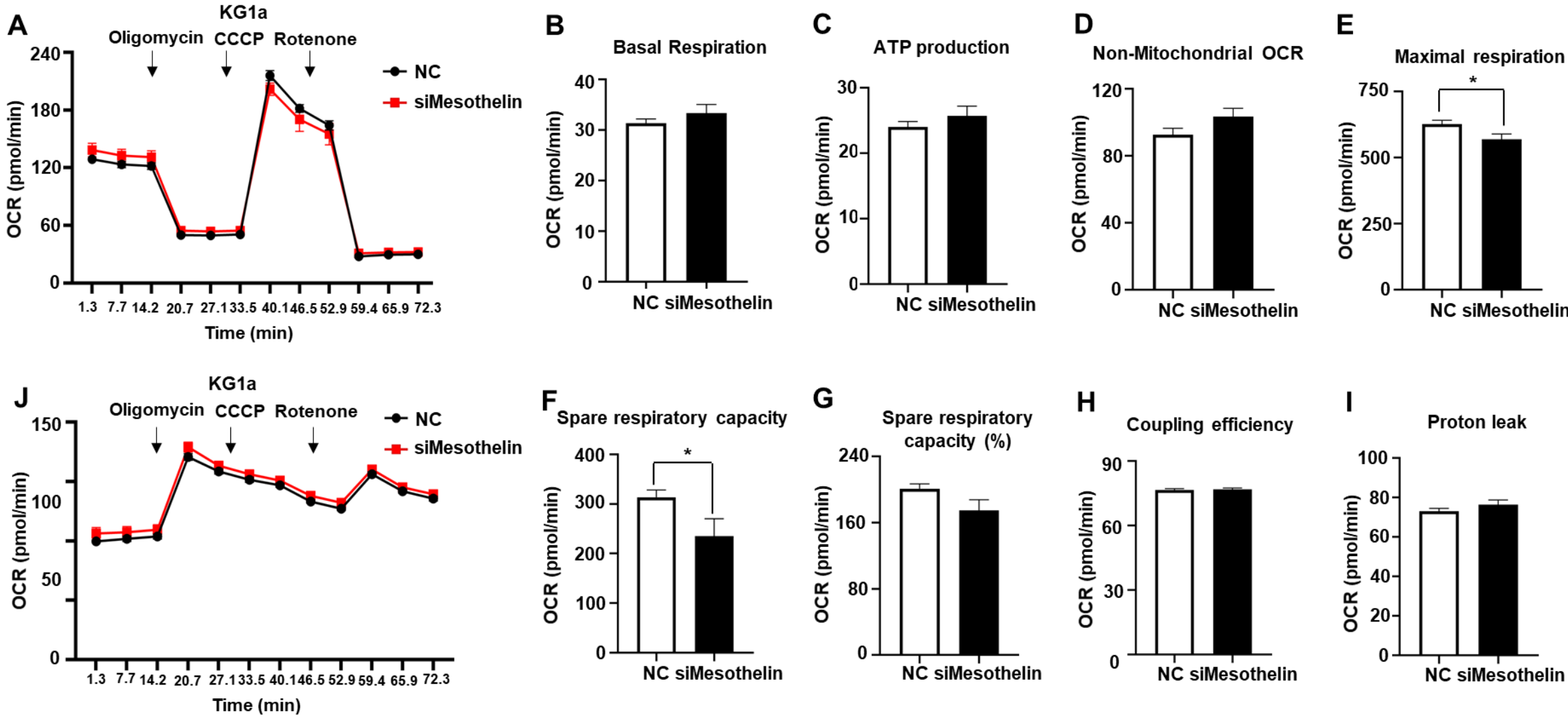

**Supplementary Figure S3**

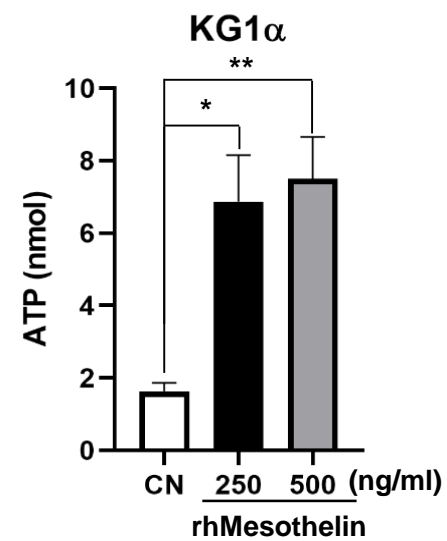

Supplement: Supplementary file 1 [file ijms-25-02140-s001.zip › ijms-2772009-supplementary.pdf]
